# Patterns and determinants of prescribed drug use among pregnant women in Adigrat general hospital, northern Ethiopia: a cross-sectional study

**DOI:** 10.1186/s12884-020-03327-7

**Published:** 2020-10-15

**Authors:** Niguse Meles Alema, Getachew Semagn, Shetey Melesse, Ephrem Mebrahtu Araya, Hagazi Gebremedhin, Desalegn Getnet Demsie, Solomon Weldegebreal Asgedom, Etasy Weldekidan

**Affiliations:** 1 Department of Pharmacy, College of Medicine and Health Sciences, Adigrat University, P.O.Box: 50, Adigrat, Ethiopia; 2 Department of Clinical Pharmacy, School of Pharmacy, College of Health Sciences, Mekelle University, Mekelle, Ethiopia

**Keywords:** FDA risk classification, Pregnancy, Drug use, Determinants, Adigrat hospital

## Abstract

**Background:**

A vigilant prescription of drugs during pregnancy can potentially safeguard the growing fetus from the deleterious effect of the drug while attempting to manage the mother’s health problems. There is a paucity of information about the drug utilization pattern in the area of investigation. Hence, this study was implemented to investigate the pattern of drug utilization and its associated factors among pregnant women in Adigrat general hospital, Northern Ethiopia.

**Methods:**

An institution-based cross-sectional study was conducted among randomly selected 314 pregnant women who attended obstetrics-gynecology and antenatal care units of the hospital. Relevant data were retrieved from the pregnant women’s medical records and registration logbook. The drugs prescribed were categorized based on the United States Food and Drug Administration (US-FDA) fetal harm classification system. Data analysis was done using SPSS version 20 statistical software. Multivariate logistic regression was employed to analyze the association of the explanatory variables with the medication use, and *p* < 0.05 was declared statistically significant.

**Results:**

The overall prescribed drug use in this study was found to be 87.7%. A considerable percentage of the study participants (41.4%) were prescribed with supplemental drugs (iron folate being the most prescribed drug) followed by antibiotics (23.4%) and analgesics (9.2%). According to the US-FDA drug’s risk classification, 42.5, 37, 13, and 7% of the drugs prescribed were from categories A, B, C, and D or X respectively. Prescribed drug use was more likely among pregnant women who completed primary [AOR = 5.34, 95% CI (1.53–18.6)] and secondary education [AOR = 4.1, 95% CI (1.16–14)], who had a history of chronic illness [AOR = 7.9, 95% CI (3.14–19.94)] and among multigravida women [AOR = 2.9, 95% CI (1.57 5.45)].

**Conclusions:**

The finding of this study revealed that a substantial proportion of pregnant women received drugs with potential harm to the mother and fetus. Reasonably, notifying health practitioners to rely on up-to-date treatment guidelines strictly is highly demanded. Moreover, counseling and educating pregnant women on the safe and appropriate use of medications during pregnancy are crucial to mitigate the burden that the mother and the growing fetus could face.

## Background

Medication use during the period of pregnancy becomes a major concern since the thalidomide incidence of the 1960s and the teratogenic effects of diethylstilbestrol discovered in 1971 [1]. Pregnancy care imposes a great challenge to both the health care providers and pregnant women because drug utilization during pregnancy may adversely affect the lives of the mothers and the growing fetus [2, 3].

In general, the use of prescription medications and other over the counter drugs should be avoided during pregnancy. However, it is unlikely to avoid all drugs during pregnancy because this may put both the mother and fetus in danger from complications of untreated acute and chronic medical disorders such as epilepsy, diabetes mellitus, thyroid disorder, severe depression, hypertension, and bronchial asthma [4, 5]. Besides, respiratory infections, headaches, constipation, nervousness, and other common complaints that occur during pregnancy may also need drug treatments. To make evidence-based healthcare decisions, different classification scheme has been established to describe the risks of medication use during pregnancy, such as the United States Food and Drug Administration (US-FDA) pregnancy risk category, the Swedish system, and the Australian Drug Evaluation Committee (ADEC) classification [6–9]. The US-FDA pregnancy risk category, which was developed in 1979, classifies drugs into five main categories: A, B, C, D, and X based on published evidence of the risks and benefits of medication use during pregnancy [6, 10]. The former US-FDA system was criticized for its oversimplification, incompetence, misinterpreted as a grading system which led to misinformation in guiding clinical practice. To address the criticism of the previous labeling system the US-FDA developed a new narrative labeling system known as Pregnancy and Lactation Labeling Rule (PLLR) in June 2015. While the new labeling improves the old format, PLLR still does not provide a definitive “yes” or “no” answer on medication safety as it requires a clinical interpretation on a case-by-case basis [11, 12]. Besides, the new narrative system has no cut-off points (complex and needs critical judgments) and cannot be studied using retrospective chart review (RCR) type of study designs [12–15]. With its some drawbacks, the old US-FDA risk category can provide a rough description of risks associated with drug use during pregnancy [9, 16] and has been widely adopted and the most well-known in drug utilization studies in both developed and developing countries [12, 13, 15, 17–19].

Several studies have been conducted in various parts of developed [20–24] and developing countries [5, 10, 25–27] to assess drug utilization patterns during pregnancy. Despite the limited information available on the safety and effectiveness of drugs in pregnancy, a large proportion of those studies reported that health care professionals prescribe and pregnant mothers consume a surprisingly large number of drugs. In developed countries, prescription drug use ranges from 27 to 93% with a substantial number of drugs from US-FDA category D and X [20]. For example, in a historical cohort study conducted in Canada, 3.9 and 5.2% of pregnant women took medications from category D and X, respectively. Similarly, the proportion of pregnant women in the USA taking category D and X medications has been estimated at 4.8 and 4.6%, respectively [24].

A small number of previous studies that have been conducted in Ethiopia reported that remarkable proportions of medications prescribed during pregnancy were unsafe. Two different studies conducted in Bahr Dar and Addis Ababa showed that 88.4 and 71.3% of the pregnant women consumed at least one prescription drug, of which 11 and 4% of them received drugs from category D or X of the US-FDA risk classification, respectively [28, 29]. Similar studies conducted in Harer and Mekelle, Ethiopia, revealed that 85.1 and 87.5% of the pregnant women used at least one prescription drug [15, 19]. In developing countries like Ethiopia, irrational use of drugs during pregnancy could be aggravated by numerous factors such as low level of educational status of the mother, lack of up-to-date information among health care providers, lack of standard drug prescribing guidelines, poor health-seeking behavior of the patients, and delayed initiation of antenatal care [28].

To facilitate the rational use of drugs in pregnancy, knowledge about the drug utilization patterns, and maternal factors affecting these patterns are crucial. But, there is a dearth of information about drug utilization patterns in pregnant women in the current study area. Therefore, this study was carried out to investigate the patterns of drug utilization and its associated factors among pregnant women in Adigrat general hospital, Northern Ethiopia. With this information, we intend to provide feedback and recommendation to health care providers, pregnant mothers, and other concerned bodies.

## Methods

### Study design, area and period

An institutional-based retrospective cross-sectional study was implemented among pregnant women who had received clinical services in obstetrics-gynecology and antenatal care units of Adigrat general hospital between November 2018 and May 2019. Adigrat hospital is found in Adigrat town, which is located 120 km away from Mekelle, the capital of the regional state of Tigrai, and 898 km to the North of Addis Ababa, the capital city of Ethiopia. It is one of the general hospitals in the Eastern zone of Tigrai that provides different health care services for patients from urban and rural surroundings. It also serves as a teaching center for Adigrat University health science students. According to the 2019 Tigrai regional office of finance and planning estimation, the Eastern zone of Tigrai has a total population of 939,739 of which 451,075 are men, and 488,664 are women.

### Study population

All medical records of pregnant women who attended obstetrics-gynecology and antenatal care units of Adigrat general hospital from November 1, 2018, to May 30, 2019, were considered as study population, and the sampling units were selected from those patient medical records.

### Sample size determination and sampling techniques

The sample size was determined using a single population proportion formula by considering the following assumption: the prevalence of drug use during pregnancy was taken as 41% from a study conducted in Mekelle [30], a confidence interval of 95% and a margin of error 5%. A correction formula was used to adjust the final sample size since the total number of pregnant women who attended the hospital during the study period was found to be 1860 (< 10,000).

Upon adding 5% contingency the final sample size was found to be 314. A systematic random sampling technique was applied to select the pregnant women’s medical charts by determining the sampling interval. Then, the first medical chart was selected by the lottery method from the patients’ medical registration logbook.

### Inclusion and exclusion criteria

All pregnant women in any trimester, attending antenatal outpatient and inpatient department in Adigrat general hospital, with or without comorbidities, who had complete data were included in the study. When pregnant women had incomplete medical records, they were excluded from the study.

### Data collection

The socio-demographic characteristics (age, religion, marital status, educational status, occupation, and residency), obstetric and medical histories (gravida, pregnancy status, and history of chronic disease), as well as drugs prescribed, were extracted from the pregnant women’s medical charts and a registration logbook using a pre-designed data collection checklist prepared by reviewing relevant pieces of literature [15, 19, 22–25, 29, 30]. Data were cleaned and checked for completeness during collection, and incomplete patient’ cards were discarded.

### Statistical analysis

Medicines prescribed to pregnant mothers were categorized according to the pharmacological and the former US-FDA risk classification systems. The collected data were coded, entered, and analyzed using Statistical Package for Social Sciences (SPSS) version 20 software. Simple descriptive statistics, including percentages, frequency, and mean ± standard deviation, were computed to summarize the data, and the result was presented in the form of tables and figures. Bivariate logistic regression was performed to assess the association between the predictor and the outcome variable, and those variables with *p* < 0.25 were transferred into multivariate logistic regression. The Hosmer and Lemeshow test was used to assess the goodness of fit of the logistic regression model [31], and *p* < 0.05 at 95% confidence interval were used to declare a statistically significant association.

### Ethical consideration

The study was ethically approved by the institutional research review board of Adigrat University. Because of the retrospective nature of the study (data were abstracted from the medical chart of pregnant mothers), we did not obtain a written or oral consent from the study participants. But, official permission was obtained from the Tigrai Regional Health Bureau and the administration of the general hospital to access their medical records after explaining the aims and purpose of the study. Besides, privacy and confidentiality were maintained throughout the study by removing any personal identifiers during the data abstraction process.

## Results

Of the 314 medical records of the pregnant women reviewed, only medical records of 277 pregnant mothers were included in the analysis making the response rate 88.2%. The remaining 37 (11.7%) medical registers of pregnant women were excluded from analysis due to the incompleteness of data.

### Socio-demographic characteristics

Of the 277 pregnant women involved in the study, 90% of them were in the age group of 20–34 years. The majority of pregnant women (79.4%) were orthodox Christians by religion, almost half of them (54.2%) were urban dwellers, and 74.7% were married and unemployed. Concerning the educational level, 43% were illiterate, 24.9% had completed primary education, 17.7% had completed secondary school, while 14.4% had a diploma and above (Table 1).

**Table 1:** Socio-demographic characteristics of pregnant women (*n* = 277) in Adigrat general hospital, Northern Ethiopia, 2019

Variables	Frequency	Percentage (%)
**Age**		
≤ 19	26	9.4
20–34	194	70
35–42	53	19.1
≥ 43	4	1.4
**Religion**		
Orthodox	220	79.4
Muslim	39	14.1
Protestant	13	4.7
Catholic	5	1.8
**Marital status**		
Single	51	18.4
Married	207	74.7
Divorced	19	6.9
**Educational status**		
Illiterates	119	43
Primary education	69	24.9
Secondary education	49	17.7
Higher education	40	14.4
**Occupation**		
Unemployed	207	74.7
Employed	70	25.3
**Residency**		
Urban	150	54.2
Rural	127	45.8

### Obstetric and medical history

As depicted in Table 2, more than half of the pregnant women (53.4%) were multigravida while the rest (46.6%) were primigravida. A substantial proportion of pregnant women (37.2%) visited the hospital in the first trimester of pregnancy, and the main reason for visiting the health facility was seeking antenatal care service (ANC). Fifty-six (20.2%) of pregnant women had a history of chronic disease. The majority (72.2%) of the pregnant women visited the hospital for a maximum of one to two times, and 78.3% of the pregnancies were planned.

**Table 2:** Obstetric and medical history of study subjects

Obstetric and medical histories	Frequency	Percentage (%)
Gravida		
Primigravidae	129	46.6
Multigravida	148	53.4
Pregnancy status		
Planned	217	78.3
Mistimed	27	9.7
Unwanted	33	11.9
Number of visits to Health facilities		
1–2 times	200	72.2
3–4 times	74	26.7
≥ 5 times	3	1.1
History of chronic Disease		
Yes	56	20.2
No	221	79.8
Reasons for a visit to the Health facilities		
ANC	246	88.8
Medical illness	29	10.5
Others	1	0.4
Time of the first visit of the health facility		
First trimester	103	37.2
Second trimester	79	28.5
Third trimester	95	34.3

### Drug use during pregnancy

Of the 277 pregnant women enrolled in this study, 243 (87.7%) of them had taken at least one prescription drug while the remaining 34 (12.3%) did not take any medication. Among those who used at least one prescription drug, 78 (32%) and 165 (68%) of them received iron folate and other class of drugs, respectively. The average number of drugs prescribed in this study was found to be one (ranging from zero to four drugs). Thirty-four different types of drugs (a total of 261) were prescribed to pregnant women (Additional file 1).

**Fig. 1:**
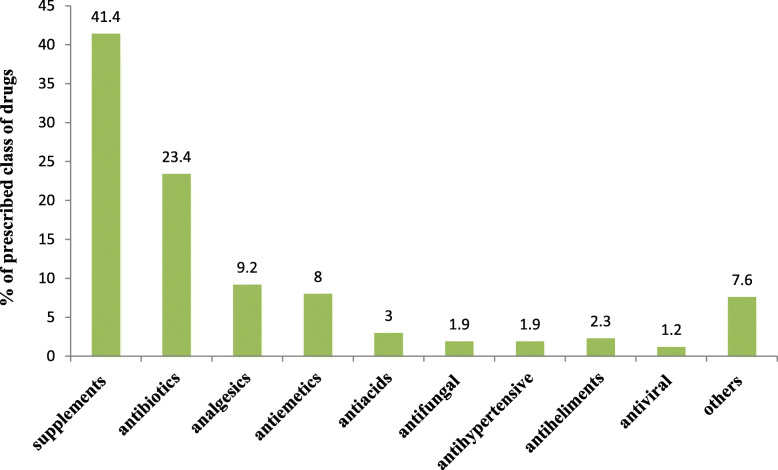
Percentage distribution of commonly prescribed class of drugs to pregnant women in Adigrat general hospital, Northern Ethiopia, 2019

A considerable proportion of pregnant mothers (41.4%) were prescribed with supplemental drugs, iron folate being the most predominantly prescribed medication from this category. Among the non-supplemental drugs, antibiotics (23.4%) were found the commonly prescribed drugs followed by analgesics (9.2%), antiemetic’s (8%), and antacids (3%), respectively (Fig. 1).

**Fig. 2:**
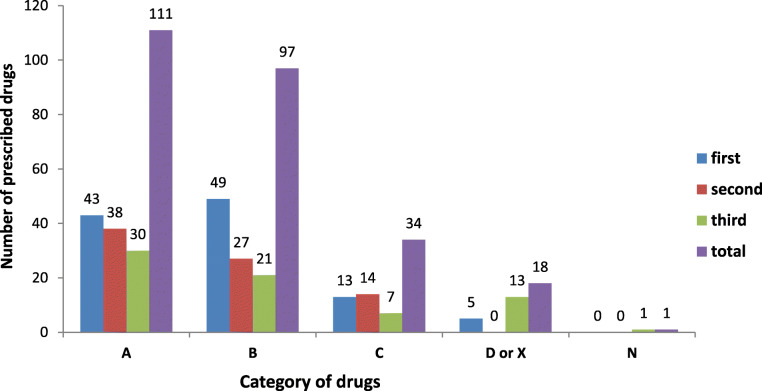
Classification of drugs prescribed according to the US-FDA risk classification and gestation age of pregnant women in Adigrat general hospital, Northern Ethiopia, 2019

The drugs prescribed during pregnancy were categorized according to the US-FDA risk classification system and their gestational age, as illustrated in Fig. 2 below. A substantial number of drugs (42.2%) were prescribed to the pregnant women in the first trimester of pregnancy, followed by second (30.2%) and third trimester (27.6%). The present study revealed that 42.5, 37, 13, and 7% of the prescribed drugs to pregnant women belong to categories A, B, C, and D or X, respectively, as per the US-FDA risk classification. Valproate sodium, ibuprofen, diclofenac, diazepam, phenobarbitone, misoprostol, and oxytocin were the drugs that have been prescribed from category D and X (Fig. 2, Table 3).

**Table 3:** Distributions of teratogenic drugs prescribed to pregnant women

Name of the drug	Indication	Frequency
Valproate sodium	Epilepsy	1
Ibuprofen^a^	Labor pain	1
Diclofenac^a^	Labor pain, headaches, and back pain	7
Diazepam	Preeclampsia	1
Phenobarbitone	Epilepsy	1
Misoprostol	Labour induction and abortion	2
Oxytocin	Labour induction	5
**Total**		**18**

^a^Considered as teratogenic when prescribed at the third trimester of pregnancy

### Factors associated with prescribed drug use during pregnancy

Multivariable logistic regression analysis showed that educational status, history of chronic disease, time of the first visit to the health facility, and gravida were significantly associated with prescribed drug use during pregnancy (Table 4). Pregnant women who completed primary [Adjusted Odds Ratio (AOR) =5.34, 95% CI (1.53–18.6)] and secondary education [AOR = 4.1, 95% CI (1.16–14)] were with high odds of being prescribed with drugs. Similarly, pregnant women with chronic illness were 7.9 [AOR = 7.92, 95% CI (3.14–19.94)] times more likely to get a drug prescribed than those with no history of medical illness. Multigravida women were 2.9 [AOR = 2.93, 95% CI (1.57–5.45)] times more likely to be prescribed with drugs compared with primigravida. However, pregnant women who visited the health facility during the first trimester had less chance of being prescribed with drugs than the pregnant women who visited the health facility in the third trimester [AOR = 0.48, 95% CI (0.23–0.98)].

**Table 4:** Factors associated with exposure to prescribed drugs among pregnant women attending Adigrat general hospital, Northern Ethiopia, 2019

Variables	COR(95% CI)	AOR(95% CI)	***P***-value
**Educational status**			
Illiterate	2.69(1.14–6.34)	2.01(0.65–6.8)	0.227
Primary education	5.9(2.56–13.61)	5.34(1.53–18.6)	0.08
Secondary education	6.69(3.07–14.55)	4.1(1.16–14)	**0.027**
Higher education	1		
**History of chronic Disease**			
Yes	7.32(3.2–16.7)	7.92(3.14–19.94)	**0.0001**
No	1		
**Time of first visit to the health facility**			
First trimester	0.3(0.17–0.54)	0.48(0.23–0.98)	**0.045**
Second trimester	0.55(0.3–1.02)	0.53(0.25–1.1)	0.088
Third trimester	1		
**Gravida**			
Primigravida	1		
Multigravida	3.93(2.4–6.45)	2.93(1.57–5.45)	**0.01**

*COR* Crude Odds Ratio; *AOR* Adjusted Odds Ratio; *CI* Confidence Interval

## Discussion

The use of prescription medications during pregnancy is prevalent, irrespective of the little information available about the safety of drugs to be used in pregnancy [1]. In this study, the overall prevalence of consumption of medications among pregnant women was 87.7%. This finding is comparable to the 87.5% in Northern Ethiopia [19] 88.4% in Bahr Dar city [28], 85.1% in Harer [15], 85.2% in Germany [20], but lower than the 96% in Nekemte City [32], 93–99% in France [20] and 93% in Iceland [33]. Moreover, the prevalence was higher than 71.3% in Addis Ababa [29], 73.2% in Cameroon [34], 79.1% in Netherlands [23], 65% in United Kingdom [24], and 56% in Canada [20].

In the present study, supplemental drugs were the most commonly prescribed category of drugs which accounted for 41.4% of the total drug used. Among the supplemental medications, iron folate utilization accounted for approximately three-quarters of the supplemental drugs, which were much higher compared to a study conducted in eight rural districts of Ethiopia where only 35.4% of the pregnant women were given iron supplements [35]. The lower utilization of iron supplements in the rural district could be partly explained due to the lower health service coverage and lack of awareness about the importance of such supplements in rural areas. In contrast to this, a study conducted in Northern Ethiopia showed a higher (95%) iron/folic acid utilization [19].

From the non-supplemental categories of drugs, antibiotics, analgesics, and antiemetic were the most frequently received drugs, which accounted for 23.4, 9.2, and 8% of the prescribed medications, respectively. This finding is in concordance with previous studies conducted in Northern Ethiopia [19], in Harer, [15] and Oman [36]. This could be enlightened by the fact that infectious diseases are common disorders in developing countries during pregnancy, and they often happen in association with fever for which analgesics are prescribed. Moreover, headaches, pain, and gastrointestinal disorders like nausea and vomiting are common complaints during the period of pregnancy, which may necessitate a drug prescription.

According to the US-FDA risk classification of drugs, the majority of the pregnant women in this study were provided with category A and category B drugs, which are considered relatively safe during pregnancy. Similar patterns of category distribution were reported from previous studies conducted in the Netherlands [23], in the Southern Tigray region, Ethiopia [19], and Swaziland [37]. However, approximately 7% of the prescribed medications in the current study belong to category D or X, which are considered potentially teratogenic when used during pregnancy. This result is higher as compared to the finding of other related studies, which reported that less than 1 % of the pregnant women received medications from this category [19, 27]. Hormonal preparations such as misoprostol (for induction of abortion and labor) and oxytocin (for induction of labor) were the drugs prescribed from category X, and their use could be justified. The drugs which were prescribed from category D were antiepileptic drugs (diazepam, phenobarbitone, and valproate sodium) and non-steroidal anti-inflammatories such as ibuprofen and diclofenac (when prescribed at the third trimester). The use of such drugs in pregnant women should be avoided unless the potential benefit outweighs their risk. Valproate sodium can cause neural tube defects [38] and maternal use of diclofenac sodium can cause premature closure and constriction of ductus arteriosus with subsequent severe pulmonary hypertension and transient right-sided hypertrophic cardiomyopathies [39]. The use of diazepam may lead to neonatal withdrawal syndrome and cardiorespiratory instability when maternal use occurred shortly before delivery [1, 12]. Thus, our study’s finding highlights the need for careful consideration while prescribing drugs to pregnant women to maximize the benefit to risk ratio. For instance, the use of valproate sodium in pregnant and childbearing women should be reserved for a type of seizure that is unresponsive to other anti-epileptic drugs [38] and the use of diclofenac in the third trimester of pregnancy could and should be replaced with a relatively safer alternative, for example, paracetamol for headaches and tramadol for back pain [12, 39, 40].

Our findings revealed that educational status, history of chronic disease, time of the first visit to the health facility, and gravida were significantly associated with prescribed drug use among pregnant women. In contrast to previous studies that reported pregnant women with higher education levels were with high odds of being prescribed with drugs [41, 42], we found that those pregnant women with lower education were more likely to be prescribed with orthodox medications. This is corroborated with the finding of other previously conducted studies [22, 33, 43]. This could be attributed to the fact that mothers with higher educational levels are less likely to take medications due to fear of side effects. They also have a better knowledge of the potential risk/ benefit of using medication during pregnancy compared to their counterparts.

Pregnant women with chronic illness were 7.9 [AOR = 7.92, 95% CI (3.14–19.94)] times more likely to consume at least one prescription medication as compared to pregnant women who had no history of medical illness. The high-level medication consumption of pregnant mothers with a chronic illness is not surprising because there might be a greater probability of prescribing medication to treat and prevent complications that may arise from the acute and chronic diseases in pregnant women with comorbidities. Our result is concordant with the finding of other previous studies conducted in Bahir Dar City and Cameroon [28, 34].

Moreover, multigravida women were 2.9 [AOR = 2.93, 95% CI (1.57–5.45)] times more likely to be prescribed with drugs as compared to primigravida. This is in agreement with the study done in Bahir Dar City administration and Cameroon [28, 34]. This could be partly explained by the fact that as the number of pregnancies increases, the risk of developing maternal complications may arise, and orthodox medications might be required for the treatment of such complications. However, our finding is in contrast to a study conducted in the Southern Tigrai region [19].

On the other hand, pregnant women who visited the health facility for the first time during the first trimester and second trimester had less chance to receive prescription drugs in comparison with the pregnant women who visited the health facility in the third trimester [AOR = 0.48, 95% CI (0.23–0.98)]. This is in agreement with the finding of previous research [22].

## Limitation of the study

Our study is not without limitations. First, the study’s cross-sectional nature limits the ability to identify the chronological occurrence of the events, even though several factors have been associated with our outcome of interest. Second, this research was conducted in one general hospital, and it may not be representative of the drug utilization pattern of the region at large. Moreover, we did not examine the drug utilization pattern of over the counter drugs and other complementary and alternative medicines which may underestimate the overall drug utilization pattern of the pregnant women in the study area. Lastly, as described above, this study only provides a rough overview of the risks of medication use during pregnancy at a hospital level. Thus, clinical consideration of appropriateness and safety of drug use during pregnancy requires further evaluation on a case-by-case basis using the new US-FDA narrative system to make an informed decision for pregnant women seeking medication therapy. Our result should be interpreted by considering those limitations.

## Conclusion

The overall prescribed drug use, including those with teratogenic potential, was found very high in Adigrat general hospital. The prescribed drug use was greater when maternal education was low, if they had a history of chronic disease, when they visited the health facility at the third trimester and when they were multiparous. Substantial proportions (7%) of the pregnant women in this study were prescribed from the US-FDA risk classification category D or X. Therefore, it is important to note that, prescribing potentially teratogenic drugs during pregnancy should be avoided as much as possible by notifying health care providers and policymakers to adapt and ensure implementation of the US-FDA new labeling system which is expected to give better guidance. Moreover, counseling and educating pregnant women on the safe and appropriate use of medications during pregnancy are crucial to mitigate the harmful effect of drugs on the mother and the growing fetus.

## Supplementary information

**Additional file 1.** Overall drugs used as per trimester and their US FDA risk classification in Adigrat general hospital Northern Ethiopia, 2019.

**Additional file 2.** Meaning of the five-letter risk classification based on the United States Food and Drug Administration, and as used in this study.

## Data Availability

All datasets on which the conclusion of this paper relies are presented with the main manuscript and additional supporting files. If additional information is required it can be obtained from the corresponding author based on a reasonable request.

## Abbreviations

ANC: Antenatal Care Service
AOR: Adjusted Odds Ratio.
PLLR: Pregnancy and Lactation Labeling Rule
RCR: Retrospective Chart Review
SPSS: Statistical Package for Social Sciences
US-FDA: United States Food and Drug Administration
