# Supplementary material for: Patterns and determinants of prescribed drug use among pregnant women in Adigrat general hospital, northern Ethiopia: a cross-sectional study
Source: BMC Pregnancy Childbirth. 2020 Oct 15;20:624. doi: 10.1186/s12884-020-03327-7 (PMC7558672; doi:10.1186/s12884-020-03327-7)
Supplement: Supplementary file 1 — Additional file 1. Overall drugs used as per trimester and their US FDA risk classification in Adigrat general hospital Northern Ethiopia, 2019. [file 12884_2020_3327_MOESM1_ESM.docx]

**Additional file 1**: Overall drugs used as per trimester and their US FDA risk classification in Adigrat general hospital Northern Ethiopia, 2019.

| **Drug name** | **1^st^-trimester frequency** | **2^nd-^trimester frequency** | **3^rd-^trimester frequency** | **Total** | **FDA risk category** |
| --- | --- | --- | --- | --- | --- |
| Augmentin | 2 | 1 | 2 | 7 | B |
| Amoxicillin | 8 | 4 | 3 | 15 | B |
| Ampicillin | 1 | 0 | 0 | 1 | B |
| Azithromycin | 3 | 3 | 2 | 8 | B |
| Bisacodyl | 1 |  | 1 | 1 | C |
| Ceftriaxone | 6 | 1 | 2 | 9 | B |
| Cephalexin | 8 | 5 | 3 | 16 | B |
| Chlorpromazine | 4 | 2 | 2 | 8 | C |
| Clotrimazole | 3 | 0 | 0 | 3 | C |
| Diazepam | 1 | 0 | 0 | 1 | D |
| Diclofenac | 0 | 2 | 7 | 9 | C/D |
| Diphenhydramine | 1 | 0 | 1 | 2 | B |
| Haemup | 4 | 2 | 0 | 6 | A |
| Hydralazine | 0 | 1 | 0 | 1 | C |
| Hydrocortisone | 1 | 1 | 0 | 2 | C |
| Ibuprofen | 0 | 0 | 1 | 1 | C/D |
| Iron folate | 26 | 28 | 24 | 78 | A |
| Ketoconazole | 1 | 1 | 0 | 2 | C |
| Magnesium sulfate | 2 | 0 | 1 | 3 | A/C |
| Mebendazole | 0 | 2 | 2 | 4 | C |
| Methyldopa | 1 | 1 | 1 | 3 | B |
| Metoclopramide | 9 | 4 | 0 | 13 | B |
| Metronidazole | 2 | 1 | 2 | 5 | B |
| Misoprostol | 1 | 0 | 1 | 2 | X |
| Multivitamins | 10 | 7 | 6 | 22 | A |
| Omeprazole | 1 | 3 | 1 | 5 | C |
| Oxytocin | 1 | 0 | 4 | 5 | X |
| Paracetamol | 7 | 4 | 3 | 14 | B |
| Phenobarbital | 1 | 0 | 0 | 1 | D |
| Ranitidine | 0 | 2 | 1 | 3 | B |
| Ringer lactate | 0 | 0 | 1 | 1 | N |
| TDF/3TC/EFV | 3 | 0 | 0 | 3 | C |
| Tinidazole | 0 | 2 | 0 | 2 | B |
| Valproate sodium | 1 | 0 | 0 | 1 | D |
| Total | 110 | 79 | 72 | 261 |  |
