# Supplementary material for: Patterns and determinants of prescribed drug use among pregnant women in Adigrat general hospital, northern Ethiopia: a cross-sectional study
Source: BMC Pregnancy Childbirth. 2020 Oct 15;20:624. doi: 10.1186/s12884-020-03327-7 (PMC7558672; doi:10.1186/s12884-020-03327-7)
Supplement: Supplementary file 2 — Additional file 2. Meaning of the five-letter risk classification based on the United States Food and Drug Administration, and as used in this study. [file 12884_2020_3327_MOESM2_ESM.docx]

**Additional file 2:** Meaning of the five-letter risk classification based on the United States Food and Drug Administration, and as used in this study **(old A-Z letters system).**

| **US-FDA pregnancy categories** | **Their meanings** |
| --- | --- |
| Category A | Adequate and well-controlled studies in pregnant women have failed to demonstrate a risk to the fetus in the first trimester of pregnancy (and there is no evidence of risk in later trimesters). |
| Category B | Animal reproduction studies have failed to demonstrate a risk to the fetus, and there are no adequate and well**-**controlled studies in pregnant women, **or** animal reproduction studies have shown adverse eﬀects, but well**-**controlled studies in pregnant women have shown no adverse eﬀects to the fetus |
| Category C | Animal reproduction studies have shown an adverse effect on the fetus and there are no adequate and well-controlled studies in humans, but potential benefits may warrant the use of the drug in pregnant women despite potential risks. |
| Category D | There is positive evidence of human fetal risk based on adverse reaction data from investigational or marketing experience or studies in humans, but potential benefits may warrant the use of the drug in pregnant women despite potential risks. |
| Category X | Contraindicated in pregnancy. Studies in animals or humans have demonstrated fetal abnormalities and/or there is positive evidence of human fetal risk based on adverse reaction data from investigational or marketing experience, and the risks involved in the use of the drug in pregnant women outweigh potential benefits. |

Sources

1. Tillett J. Medication use during pregnancy and lactation: the new FDA drug labeling. The Journal of perinatal & neonatal nursing. 2015 Apr 1;29(2):97-9.
2. [Content and Format of Labeling for Human Prescription Drug and Biological Products; Requirements for Pregnancy and Lactation Labeling](http://frwebgate.access.gpo.gov/cgi-bin/getdoc.cgi?dbname=2008_register&docid=fr29my08-33.pdf) (Federal Register/Vol. 73, No. 104/Thursday, May 29, 2008)
